# HER2 exon 27 mutations predict worse survival of breast cancer patients, especially in HER2‐negative patients

**DOI:** 10.1002/cam4.1236

**Published:** 2017-10-26

**Authors:** Pilei Si, Tao Chen, Bin Fang, Jiabing Yao, Gaoxiu Liu, Haijun Chen, Baoping Zhai, Wentao Li

**Affiliations:** ^1^ Department of Breast Surgery Henan Provincial People's Hospital Zhengzhou 450003 China; ^2^ Department of Breast Surgery Zhengzhou University People's Hospital Zhengzhou 450003 China; ^3^ Henan University Medical School Henan University People's Hospital Zhengzhou 450003 China

**Keywords:** Breast cancer, HER2, mutation, prognosis, survival

## Abstract

The aims of this study were to assess the prognostic value of the HER2 exon 27 mutations in breast cancer patients. Genomic DNA was isolated from peripheral blood leukocytes, and then HER2 exon 27 mutations were detected by direct sequencing. Survival curves were estimated by Kaplan–Meier curves and the differences between the curves were compared by log‐rank tests. A total cohort of 892 female patients with operable primary breast cancer was included in this study. The median follow‐up was 47 months. Of these 892 patients, 3.7% (33/892) had HER2 exon 27 mutations. Patients with the HER2 exon 27 mutations had a significant worse recurrence‐free survival (RFS, unadjusted hazard ratio [HR] 2.42; 95% CI: 1.05–5.58; *P *= 0.032) and distant recurrence‐free survival (DRFS, unadjusted HR 2.81; 95% CI: 1.21–6.50; *P *= 0.012) than the patients with the wild‐type exon 27. Among the 673 patients with negative HER2 expression, 24 mutants were found. Patients with the HER2 mutations showed a worse RFS (unadjusted HR 5.08; 95% CI: 2.14–12.02; *P *< 0.001) and DRFS (unadjusted HR 5.62; 95% CI: 2.36–13.40; *P *< 0.001) than those patients with the wild‐type exon 27. Furthermore, the mutations remained as unfavorable independent predictors for RFS and DRFS. Breast cancer patients with HER2 exon 27 mutations have a worse survival, especially in HER2‐negative patients. HER2‐negative patients with HER2 exon 27 mutations are potential subgroup of breast cancer patients benefiting from HER2‐targeted therapy in future.

## Introduction

Breast cancer is a heterogeneous disease and the most common cancer in women worldwide, both in the developed and developing countries. The HER2 gene (also known as erbB‐2 or neu), located on chromosome 17q21, belongs to the family of epidermal growth factor receptor (EGFR). It encodes a transmembrane glycoprotein (p185) with tyrosine kinase activity, which is closely related to EGFR, and plays an important role in the regulation of cell growth, differentiation, and invasion 1. HER2 overexpression due to gene amplification is a recognized negative prognostic factor in breast cancer 2, 3, 4, 5. HER2‐targeted therapy significantly improves the survival of breast cancer patients with HER2 overexpression 6, 7, 8, 9.

HER2 protein consists of four domains: extracellular domain, transmembrane domain, tyrosine kinase domain, and the carboxy tail 10. The tail coding region of HER2 protein is a largely unstructured domain and plays a critical role in the regulation of the enzyme activity of the kinase 11, 12, 13, 14. We hypothesized that alteration of the tail coding region may affect the function of HER2 protein, and therefore influence the prognosis of breast cancer patients. To date, however, few studies are available in such topic.

Exon 27 is the last exon of HER2 gene and is located in the tail coding region. Missense mutation in exon 27 may release the inhibitory effects on the carboxy tail, autophosphorylate a series of tyrosine kinases in trans fashion, initiate signaling cascades, change the biological behavior of tumor cells, promote tumor progression, and deteriorate the prognosis of patients 11, 15, 16, 17, 18. Therefore, seeking to determine the prognostic effects of HER2 exon 27, we characterized its mutation status in a cohort of 892 women with operable primary breast cancer and explored the association between the mutations and survival in this cohort.

## Materials and Methods

### Patients

A total of 960 female patients with operable primary breast cancer (stages I–III) were treated at Breast Surgery Department, Henan Provincial People's Hospital from October 2011 to June 2016. Of these 960 patients, 68 were excluded due to poor quality of DNA samples or loss of follow‐up. Consequently, 892 patients were included in this study. Ages at diagnosis of the patients ranged from 20 to 83 years with a median age of 50 years. Tumor size was defined as the maximum diameter measured by ultrasound at the time of diagnosis. Tumor stage was determined according to the tumor‐node‐metastasis classification of the Union International Contre Le Cancer. All clinical characteristics such as tumor size, tumor grade, estrogen receptor (ER) status, progesterone receptor (PR) status, HER2 status, lymph node status, and adjuvant therapy were retrieved from medical records (Table 1). All procedures performed in studies involving human participants were in accordance with the ethical standards of the institutional and/or national research committee and with the 1964 Helsinki declaration and its later amendments or comparable ethical standards. Informed consent was obtained from all individual participants included in the study. This study was approved by the Research and Ethics Committee of Henan Provincial People's Hospital.

**Table 1:** Associations between HER2 exon 27 mutations and clinicopathological characteristics

Characteristics	Overall	Wild type *N* (%)	Mutations *N* (%)	*P*
Total	892	859 (96.3)	33 (3.7)	
Age at diagnosis				
≤40 year	156	143 (92.9)	11 (7.1)	0.013
>40 year	736	716 (97.0)	22 (3.0)	
Tumor size				
≥2 cm	458	438 (95.6)	20 (4.4)	0.25
<2 cm	413	401 (97.1)	12 (2.9)	
Tumor grade				
I	125	119 (95.2)	6 (4.8)	0.51
II	546	527 (96.5)	19 (3.5)	
III	127	120 (94.5)	7 (5.5)	
Unknown	94			
Lymph nodes				
Positive	325	316 (97.2)	9 (2.8)	0.38
Negative	540	519 (96.1)	21 (3.9)	
Unknown	27			
ER expression				
Positive	632	606 (95.9)	26 (4.1)	0.39
Negative	243	236 (97.1)	7 (2.9)	
Unknown	17			
PR expression				
Positive	569	545 (95.8)	24 (4.2)	0.34
Negative	306	297 (97.1)	9 (2.9)	
Unknown	17			
HER2 expression				
Positive	197	194 (95.6)	3 (4.4)	0.57
Negative	673	649 (96.4)	24 (3.6)	
Unknown	22			
Adjuvant therapy				
C	347	336 (97.4)	9 (2.6)	0.48
E	166	158 (95.2)	8 (4.8)	
C + E	310	296 (95.5)	14 (4.5)	
No therapy	69	67 (97.1)	2 (2.9)	

ER, estrogen receptor; PR, progesterone receptor; HER2, human epidermal growth factor receptor 2; C, chemotherapy; E, endocrine therapy; C + E, chemotherapy plus endocrine therapy.

### Mutation detection

Genomic DNA was isolated from peripheral blood leukocytes using a phenol‐chloroform method and then the HER2 gene was amplified by polymerase chain reaction (PCR) in a thermocycler. The mutations of HER2 exon 27 was detected by Sanger sequencing. The PCR was performed in 20 *μ*L of solution containing 25–30 ng of template DNA, 2.5 mmol/L Mg^2+^, 1.0× PCR buffer, 0.8 mmol/L dNTP, 0.5 *μ*mol/L forward and reverse primers, and 1.25 unit DNA polymerase. The reaction condition was initially 94°C for 5 min to activate Taq DNA polymerase, followed by 35 cycles of denaturation at 94°C for 30 sec, annealing at 64°C for 30 sec and extension at 72°C for 45 sec, with final elongation for 10 min. The primer sequences were: forward 5′‐CCTGCCCTCTGAGACTGATG‐3′, and reverse 5′‐ GTTCCTCTTCCAACGAGGCT ‐3′. All fragments were sequenced using automated sequencer.

### Assessment of ER/PR/HER2 expression

The ER, PR, and HER2 expression status of each patient was obtained from the pathology reports. The expression status was determined by immunohistochemistry (IHC) according to a standard method. IHC is the best way to determine the state of hormone receptors and ≥1% of the positive nucleotides are positive for hormone receptors. Tissues were fixed in 10% neutral buffered formalin for at least 24 h; 4–12 *μ*m sections were prepared on the microtome and placed on clean, positively charged microscope slides. Slides were washed three times for 5 min in xylene. Rehydration: (1) Slides were washed three times for 3 min in 100% alcohol; (2) Slides were washed two times for 3 min in 95% alcohol. (3) Slides were washed two times for 3 min in 80% alcohol. (4) Slides were rinsed for 5 min in running distilled water. Antigen retrieval: (1) Slides were heated in 10 mmol/L sodium citrate buffer, pH 6.0 at 95–100°C for 20 min. (2) The slides were removed from heat and allowed to stand at room temperature in buffer for 20 min. (3) The slides were rinsed in TBST for 1 min. Immunostaining: (1) Blocking solution of 100 *μ*L per slide was added and incubated for 20–30 min at room temperature. (2) The blocking solution was drained from the slides after which 100 *μ*L per slide of diluted primary antibody at recommended concentration was applied and incubated for 45 min at room temperature or overnight at 4°C. (3) The slides were washed in 1× TBST four times for 5 min. (4) Diluted conjugated secondary antibody of 100 *μ*L per slide was applied and incubated for 30 min at room temperature. (5) The slides were washed in 1× TBST four times for 5 min. (6) Color development was applied for (i.e., enzyme substrate) 30 min. (7) Slides were washed in 1X TBST four times for 5 min. (8) Slides were washed in distilled water for 1 min. Dehydrate and mount slides: (1) Slides were washed in two changes of 80% alcohol, 1 min each. (2) Slides were washed in two changes of 95% alcohol, 1 min each. (3) Slides were washed in three changes of 100% alcohol, 1 min each. (4) Slides were washed in three changes of xylene 1 min each. (5) Coverslip was applied. A score of 0 and 1+ was considered HER2 negative and score of 3+ was considered HER2 positive; a score of 2+ was further evaluated by fluorescence in situ hybridization (FISH) 19. FISH positive was based on: (1) Dual‐probe HER2/CEP17 ratio ≥2.0 with an average HER2 copy number ≥4.0; (2) Dual‐probe HER2/CEP17 ratio <2.0 with an average HER2 copy number ≥6.0 signals/cell. FISH negative was based on: (1) Dual‐probe HER2/CEP17 ratio <2.0 with an average HER2 copy number <4.0 signals/cell.

### Statistical analysis

The statistical analysis was performed using the SPSS software package 20.0. The association between the mutations and clinicopathologic characteristics was evaluated using Pearson's Chi‐squared test. Recurrence‐free survival (RFS) and Distant recurrence‐free survival (DRFS) curves were evaluated by Kaplan–Meier analysis and the differences between the curves were compared by log‐rank tests. RFS was defined as the time from the date of pathological diagnosis to the date when locoregional recurrence or metastases, distant metastases or death from breast cancer. DRFS was defined as the time from diagnosis to the occurrence of distant metastasis or death from breast cancer. Patients who did not relapse were censored at the time of last follow‐up. Furthermore, multivariate survival analysis was done to identify independent prognostic variables in the total cohort and HER2‐negative breast cancer patients, respectively. The HER2 exon 27 mutations was variable, and clinical features such as age at diagnosis, tumor size, tumor grade, lymph node status, ER expression, PR expression, HER2 expression, and adjuvant therapy were covariates. All statistical tests were two sided, and *P* < 0.05 was considered statistically significant.

## Results

### Patient characteristics

The clinicopathological characteristics of 892 patients are shown in Table 1. The mutations in exon 27 was determined in these 892 patients and sequence graphs are shown in Figure S1. Finally, 33 patients with exon 27 mutations were identified and the frequency was 3.7% (33/892). Among these 33 breast cancer patients with exon 27 mutations, 9 patients were HER2 positive and the other 24 patients negative, 3 mutants were c.3430G>C p.D1144H, 3 mutants were c.3436C>T p.R1146W, 9 mutants were c.3574‐3576delGAG p.1192delE, 4 mutants were c.3662A>G p.Y1221C, 4 mutants were c.3688C>T p.R1230W, 5 mutants were c.3689G>C p.R1230P, 1 mutant was c.3725C>T p.T1242M, 1 mutant was c.3647C>A p.A1216D, and 3 mutants were c.3458G>C p.R1153Q. The frequency of HER2 exon 27 mutations in patients younger than 40 years was significantly higher than that in patients older than 40 years (*P *= 0.013). No significant associations were found between mutations and tumor size, tumor grade, lymph node metastasis, ER status, PR status, HER2 status, and adjuvant therapy in the total 892 patients (Table 1).

### Association between mutations and survival

**Figure 1:**
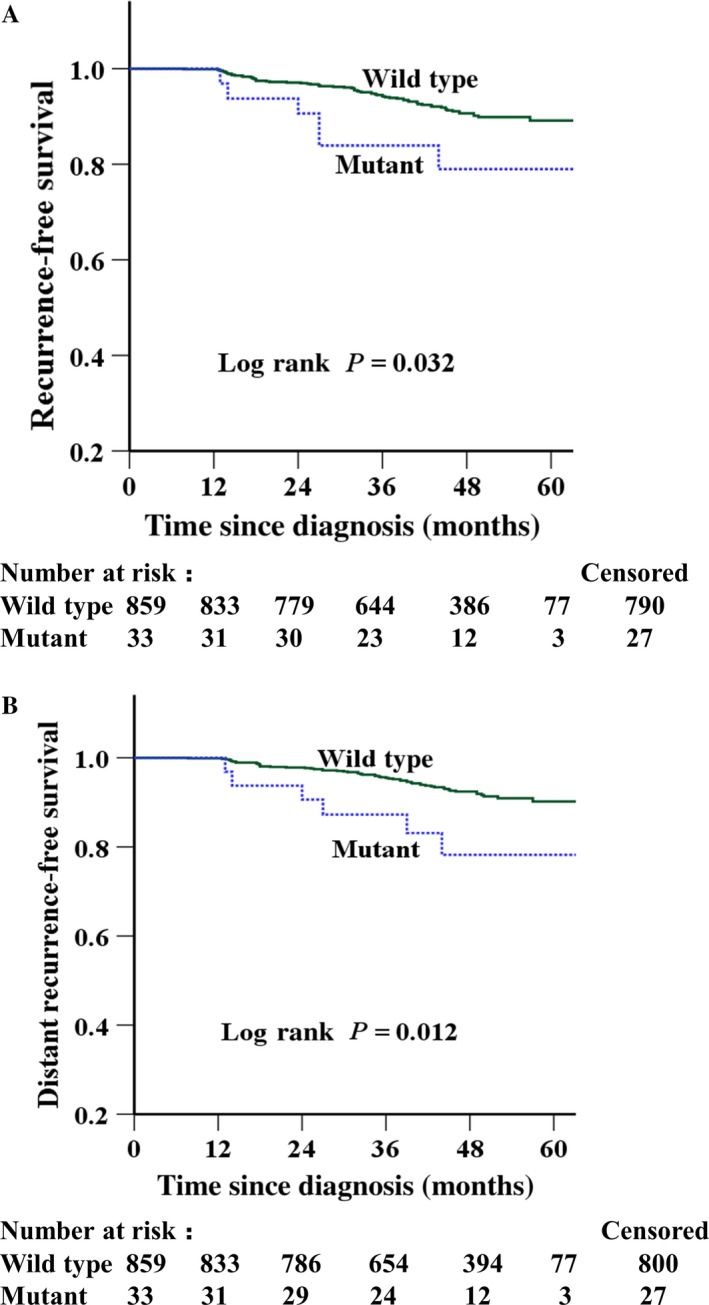
Kaplan–Meier survival curves of (A) recurrence‐free survival (RFS) and (B) distant recurrence‐free survival (DRFS) in the entire cohort of 892 patients.

The median follow‐up time for all 892 patients was 47 months (ranged from 4 to 63 months). The estimated 5‐year RFS and DRFS of the 892 patients were 88.8% (95% confidence interval [CI]: 86.1–91.5%) and 89.8% (95% CI: 87.1–92.5%), respectively. The estimated 5‐year RFS and DRFS of the 33 mutant patients were 79.0% (95% CI: 63.7–94.3%) and 78.2% (95% CI: 62.3–94.1%), respectively. Patients with the HER2 exon 27 mutations had a significant worse RFS (unadjusted hazard ratio [HR] 2.42; 95% CI: 1.05–5.58; *P *= 0.032) and DRFS (unadjusted HR 2.81; 95% CI: 1.21–6.50; *P *= 0.012) than the patients with wild‐type genotype (Table 2 and Fig. 1). Furthermore, multivariate analysis revealed that the mutations remained as a significant unfavorable factor for RFS (adjusted HR 2.81; 95% CI: 1.08–7.29; *P *= 0.035) and DRFS (adjusted HR 3.60; 95% CI: 1.37–9.47; *P *= 0.009) after adjustment for age, tumor size, tumor grade, lymph node metastasis, ER, PR, HER2, and adjuvant therapy (Table 3).

**Table 2:** Univariate analyses of RFS and DRFS in 892 breast cancer patients

Variables	RFS		DRFS	
	HR (95% CI)	*P*	HR (95% CI)	*P*
Exon 27				
(Mutant vs. Wild type)	2.42 (1.05–5.58)	0.032	2.81 (1.21–6.50)	0.012
Age at diagnosis				
(≤40 year vs. >40 year)	2.30 (1.41–3.76)	0.001	2.12 (1.24–3.62)	0.006
Tumor size				
(≥2 cm vs. <2 cm)	1.61 (0.99–2.61)	0.06	1.76 (0.96–3.22)	0.07
Tumor grade				
(III vs. I or II)	1.59 (0.89–2.83)	0.12	1.43 (0.66–3.10)	0.36
Lymph nodes				
(Positive vs. negative)	1.83 (1.15–2.91)	0.010	1.85 (1.12–3.04)	0.016
ER expression				
(Negative vs. positive)	1.68 (1.06–2.68)	0.029	1.78 (1.08–2.93)	0.024
PR expression				
(Negative vs. positive)	1.84 (1.17–2.91)	0.009	2.07 (1.27–3.39)	0.004
HER2 expression				
(Positive vs. negative)	2.28 (1.44–3.61)	<0.001	1.98 (1.20–3.26)	0.008
Adjuvant therapy				
(Yes vs. none)	1.92 (0.96–3.86)	0.07	2.00 (0.95–4.19)	0.07

RFS, recurrence‐free survival; DRFS, distant relapse‐free survival; HR, hazard ratio; CI, confidence interval; ER, estrogen receptor; PR, progesterone receptor; HER2, human epidermal growth factor receptor 2.

**Table 3:** Multivariate analyses of RFS and DRFS in 892 breast cancer patients

Variables	RFS		DRFS	
	HR (95%CI)	*P*	HR (95%CI)	*P*
Exon 27				
(Mutant vs. Wild type)	2.81 (1.08–7.29)	0.035	3.60 (1.37–9.47)	0.009
Age at diagnosis				
(≤40 year vs. >40 year)	1.09 (0.47–2.53)	0.84	1.06 (0.44–2.54)	0.90
Tumor size				
(≥2 cm vs. <2 cm)	2.00 (1.06–3.79)	0.033	1.97 (0.98–3.94)	0.06
Tumor grade				
(III vs. I or II)	1.34 (0.65–2.76)	0.43	1.43 (0.66–3.10)	0.36
Lymph nodes				
(Positive vs. negative)	3.40 (1.84–6.29)	<0.001	3.81 (1.95–7.45)	<0.001
ER expression				
(Negative vs. positive)	2.13 (1.21–3.76)	0.009	2.01 (1.08–3.75)	0.028
PR expression				
(Negative vs. positive)	2.91 (1.25–6.76)	0.013	2.33 (0.99–5.49)	0.05
HER2 expression				
(Positive vs. negative)	1.52 (0.72–3.19)	0.27	2.14 (0.90–5.10)	0.08
Adjuvant therapy				
(Yes vs. none)	2.31 (0.90–5.93)	0.08	2.79 (1.07–7.24)	0.036

RFS, recurrence‐free survival; DRFS, distant relapse‐free survival; HR, hazard ratio; CI, confidence interval; ER, estrogen receptor; PR, progesterone receptor; HER2, human epidermal growth factor receptor 2.

### Association between mutations and survival in HER2‐negative breast cancer

**Figure 2:**
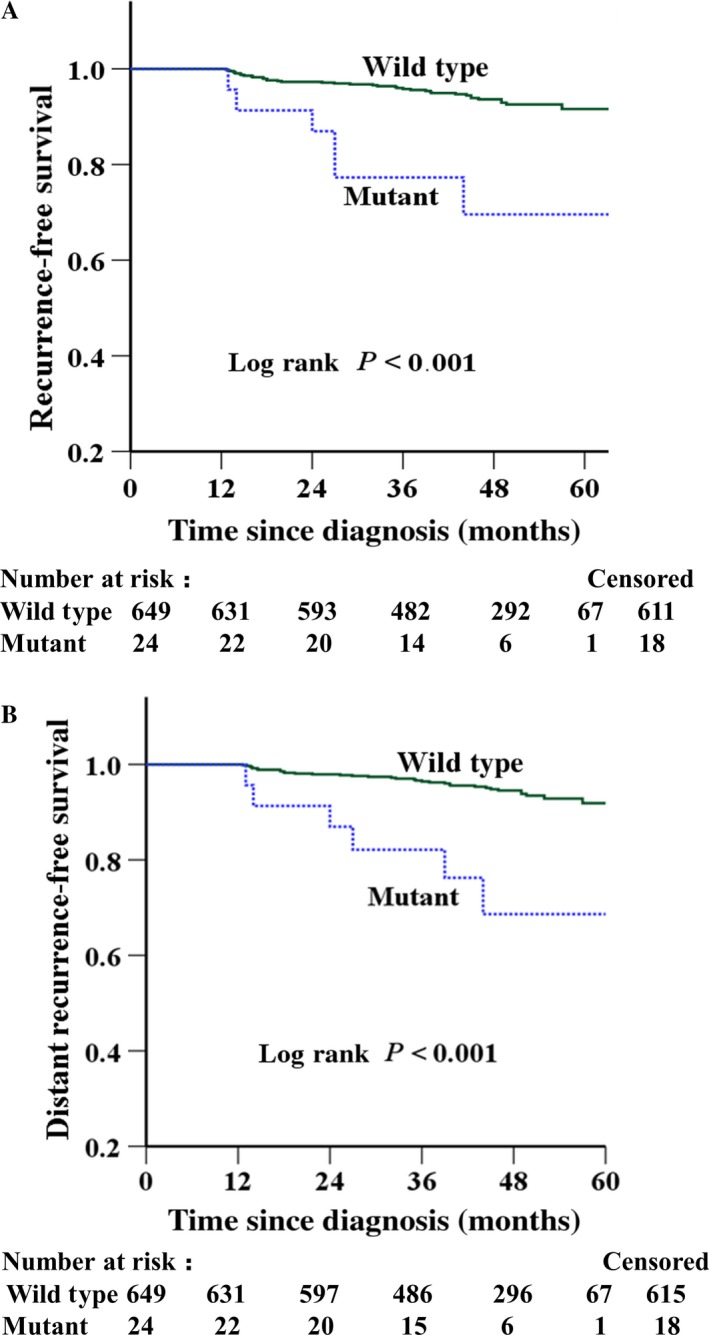
Kaplan–Meier survival curves of (A) recurrence‐free survival (RFS) and (B) distant recurrence‐free survival (DRFS) in the 673 HER2‐negative patients.

The expression levels of HER2 were evaluated in 870 patients. Of these patients, 197 (22%) patients were HER2 positive, 673 (75%) patients HER2 negative, and the immunostaining information for the other 22 (3%) patients were unavailable. And then, we analyzed the association between the mutations and survival of patients with HER2‐positive and ‐negative tumors, respectively. Among the 673 HER2‐negative patients, the exon 27 mutations of HER2 was observed in 24 patients. Patients with HER2 mutations showed a worse RFS (unadjusted HR 5.08; 95% CI: 2.14–12.02; *P *< 0.001) and DRFS (unadjusted HR 5.62; 95% CI: 2.36–13.40; *P *< 0.001) than those patients with the wild‐type genotype (Table 4 and Fig. 2). Furthermore, multivariate analysis revealed that the mutations remained as a significantly unfavorable factor for RFS (adjusted HR 6.52; 95% CI: 2.32–18.31; *P *< 0.001) and DRFS (adjusted HR 8.09; 95% CI: 2.83–23.18; *P *< 0.001) (Table 5) after adjustment for age, tumor grade, tumor size, lymph node metastasis, ER, PR, and adjuvant therapy in those breast cancer patients without HER2 expression.

**Table 4:** Univariate analyses of RFS and DRFS in 673 HER2‐negative breast cancer patients

Variables	RFS		DRFS	
	HR (95%CI)	*P*	HR (95%CI)	*P*
Exon 27				
(Mutant vs. Wild type)	5.08 (2.14–12.02)	<0.001	5.62 (2.36–13.40)	<0.001
Age at diagnosis				
(≤40 year vs. >40 year)	2.60 (1.38–4.90)	0.03	2.38 (1.21–4.69)	0.012
Tumor size				
(≥2 cm vs. <2 cm)	2.11 (1.10–4.06)	0.025	2.02 (1.02–4.00)	0.045
Tumor grade				
(III vs. I or II)	1.14 (0.45–2.92)	0.78	1.31 (0.51–3.38)	0.58
Lymph nodes				
(Positive vs. Negative)	2.47 (1.35–4.53)	0.004	2.33 (1.24–4.39)	0.009
ER expression				
(Negative vs. Positive)	1.23 (0.62–2.43)	0.56	1.41 (0.70–2.81)	0.34
PR expression				
(Negative vs. Positive)	1.67 (0.91–3.06)	0.10	1.95 (1.04–3.65)	0.037
Adjuvant therapy				
(Yes vs. none)	1.58 (0.56–4.41)	0.39	1.77 (0.63–4.99)	0.28

RFS, recurrence‐free survival; DRFS, distant recurrence‐free survival; HR, hazard ratio; CI, confidence interval; ER, estrogen receptor; PR, progesterone receptor.

**Table 5:** Multivariate analyses of RFS and DRFS in 673 HER2‐negative breast cancer patients

Variables	RFS		DRFS	
	HR (95% CI)	*P*	HR (95% CI)	*P*
Exon 27				
(Mutant vs. Wild type)	6.52 (2.32–18.31)	<0.001	8.09 (2.83–23.18)	<0.001
Age at diagnosis				
(≤40 year vs. >40 year)	1.34 (0.48–3.74)	0.58	1.28 (0.50–3.27)	0.61
Tumor size				
(≥2 cm vs. <2 cm)	1.88 (0.93–3.81)	0.08	1.79 (0.85–3.75)	0.12
Tumor grade				
(III vs. I or II)	1.27 (0.54–3.00)	0.59	1.12 (0.39–3.23)	0.84
Lymph nodes				
(Positive vs. Negative)	3.55 (1.69–7.47)	0.001	3.73 (1.70–8.22)	0.001
ER expression				
(Negative vs. Positive)	2.18 (1.03–4.62)	0.042	1.95 (0.86–4.43)	0.11
PR expression				
(Negative vs. Positive)	3.16 (1.25–7.97)	0.015	3.84 (1.96–10.09)	0.006
Adjuvant therapy				
(Yes vs. none)	1.62 (0.46–5.65)	0.45	1.78 (0.49–6.39)	0.38

RFS, recurrence‐free survival; DRFS, distant recurrence‐free survival; HR, hazard ratio; CI, confidence interval; ER, estrogen receptor; PR, progesterone receptor.

Among the HER2‐positive breast cancer patients, only three patients with HER2 exon 27 mutations were found. Due to the underlying major bias, statistical analysis was not conducted.

## Discussion

To the best of our knowledge, this study is the first to investigate the association between the mutations of HER2 carboxy tail region and survival in breast cancer patients. Significant association was found between the mutations and unfavorable survival in the entire cohort of 892 breast cancer patients and in HER2‐negative patients. Patients with these exon 27 mutations were associated with a worse RFS and DRFS than those patients with wild‐type genotypes in the total cohort and in the HER2‐negative breast cancer patients. Furthermore, these mutations remained as an independent unfavorable factor for RFS and DRFS in breast cancer patients.

HER2 gene amplification and protein overexpression have been associated with increased risk of unfavorable survival compared with patients of HER2‐negative expression 2, 3, 4, 5. Treatment with HER2‐targeted monoclonal antibody has significantly improved the survival of patients with HER2‐positive breast cancer 6, 7, 8, 9. Although patients with HER2‐negative breast cancer generally have a more favorable survival than patients with HER2‐positive disease, a minority of HER2‐negative patients may still recur locally or distantly after treatment 20. In addition, efficient targeted therapy for HER2‐negative breast cancer is unavailable at present.

HER2 gene mutations were predominantly distributed in HER2‐negative patients 21. With the rapid development of precision medicine, HER2 mutations may be one of the most potential treatment targets for breast cancer, especially for the HER2‐negative breast cancer. Research of the HER2 gene mutations may determinate the patients with relatively higher risk from the entire HER2‐negative breast cancer patients and open up a new pointcut to treat HER2‐negative breast cancer precisely. Recently, a phase II study of neratinib for breast cancer patients with HER2 mutations but without HER2 expression has been instituted (NCT01670877). Another open‐label, phase II study about neratinib therapy in breast cancer has been initiated as well (NCT01953926).

In conclusion, breast cancer patients with HER2 exon 27 mutations have a worse survival, especially in HER2‐negative patients. The HER2 exon 27 mutations can differentiate the subgroup with worse survival from HER2‐negative patients and might be a novel therapeutic target for HER2‐negative patients in future. To confirm our findings, further independent studies are warranted.

## Conflict of Interest

The authors have no conflict of interest to declare.

## Supporting information


**Figure S1.** HER2 exon 27 mutations detected by DNA direct sequencing.
