# Supplementary material for: HER2 exon 27 mutations predict worse survival of breast cancer patients, especially in HER2‐negative patients
Source: Cancer Med. 2017 Oct 26;6(12):2832–9. doi: 10.1002/cam4.1236 (PMC5727320; doi:10.1002/cam4.1236)
Supplement: Supplementary file 1 — Figure S1. HER2 exon 27 mutations detected by DNA direct sequencing. [file CAM4-6-2832-s001.docx]

**Supporting information**

**Appendix Figure 1** HER2 exon 27 mutations detected by DNA direct sequencing.

**A**

**
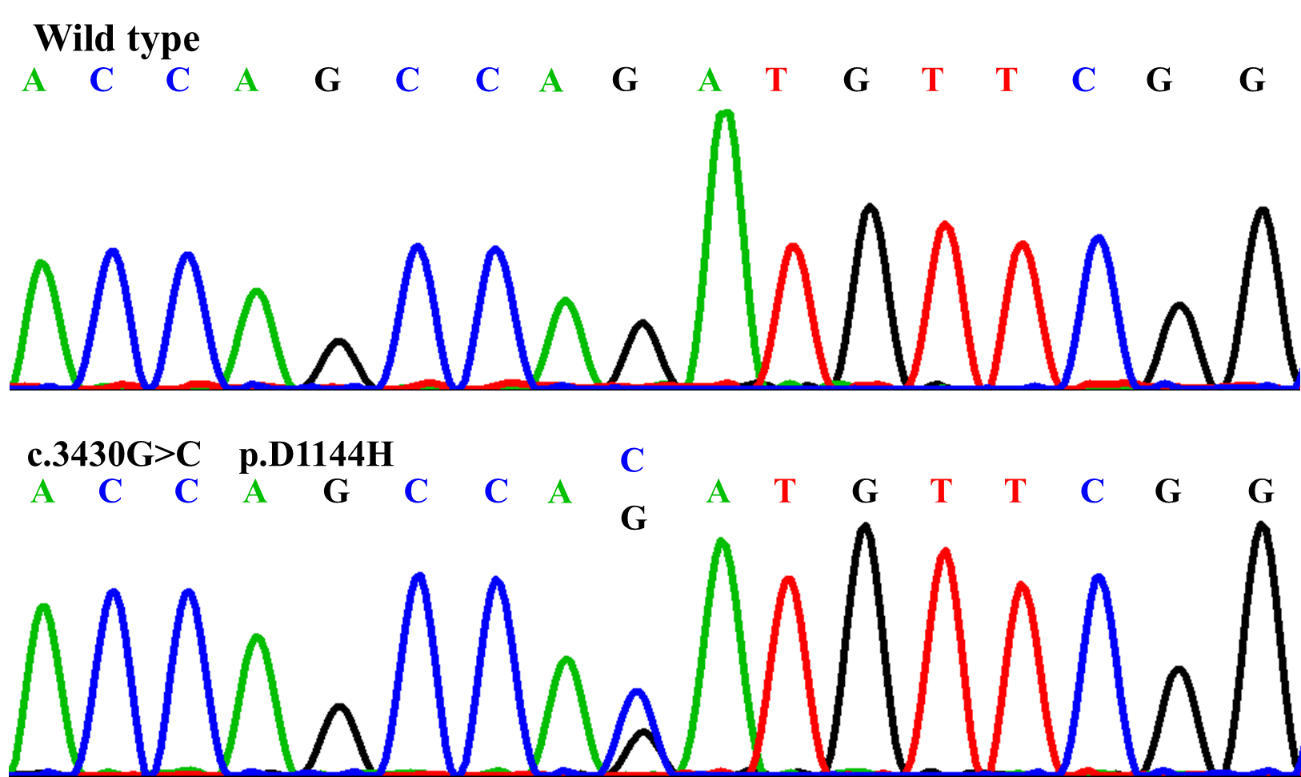
**

**B**


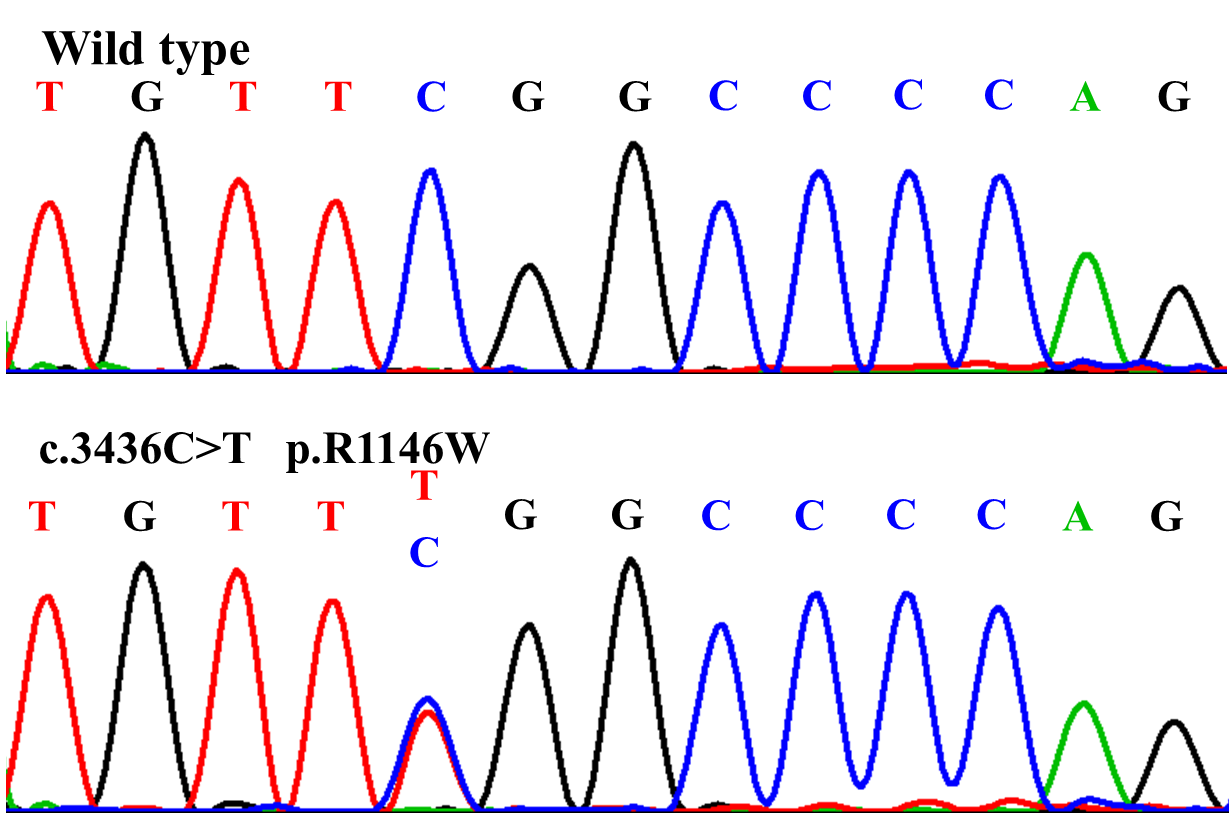


**C**

**
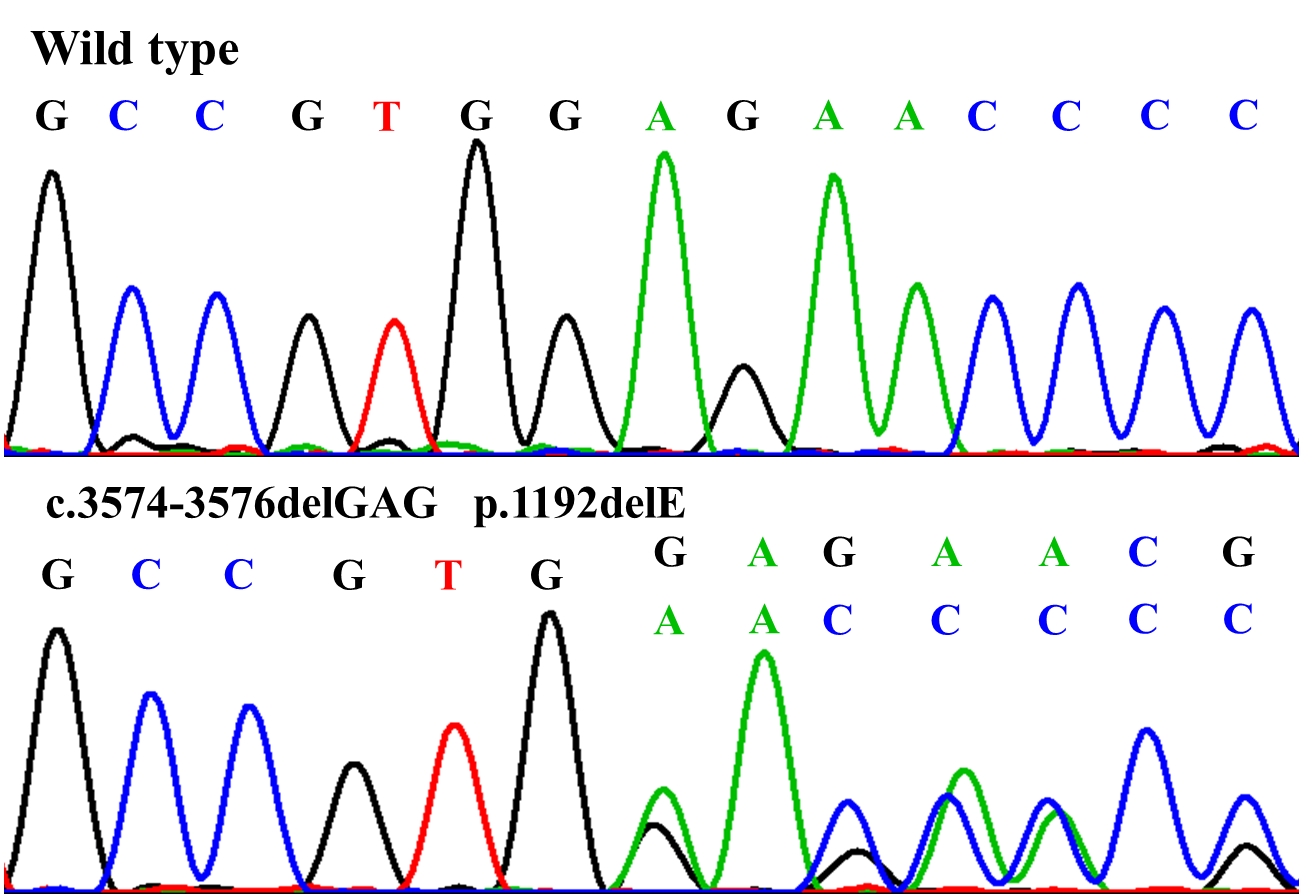
**

**D**

**
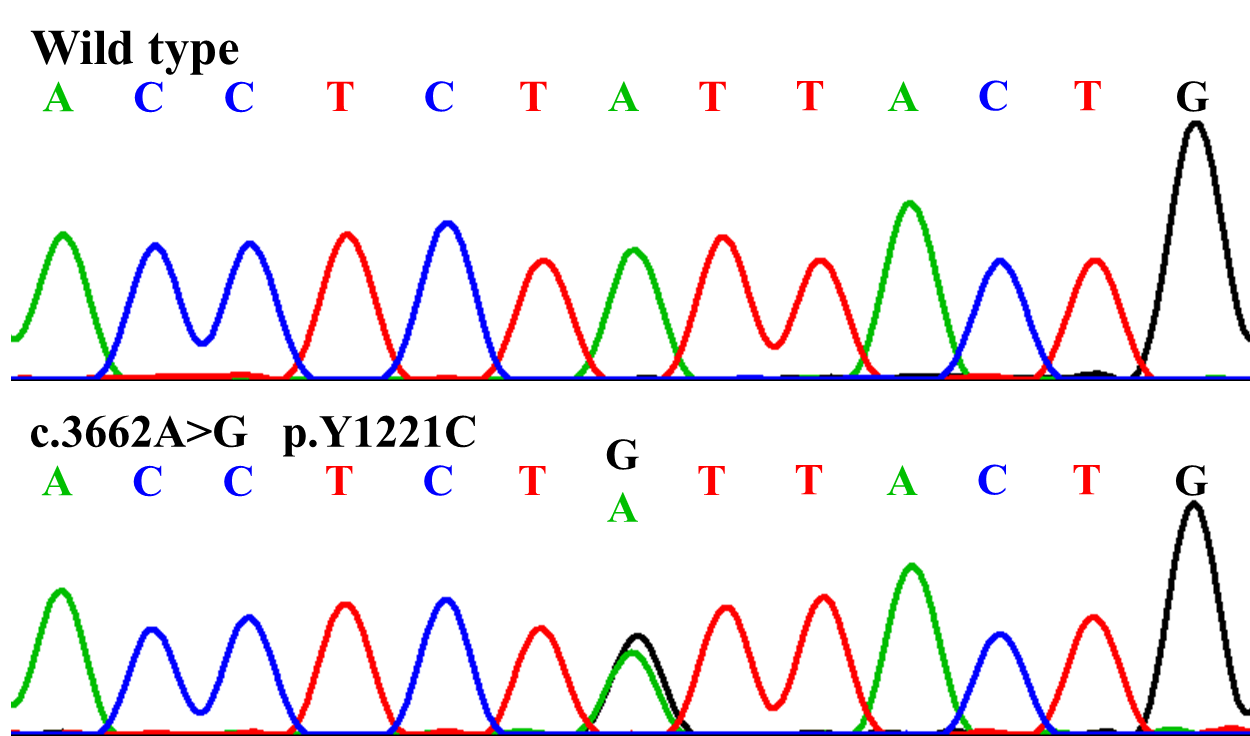
**

**E**

**
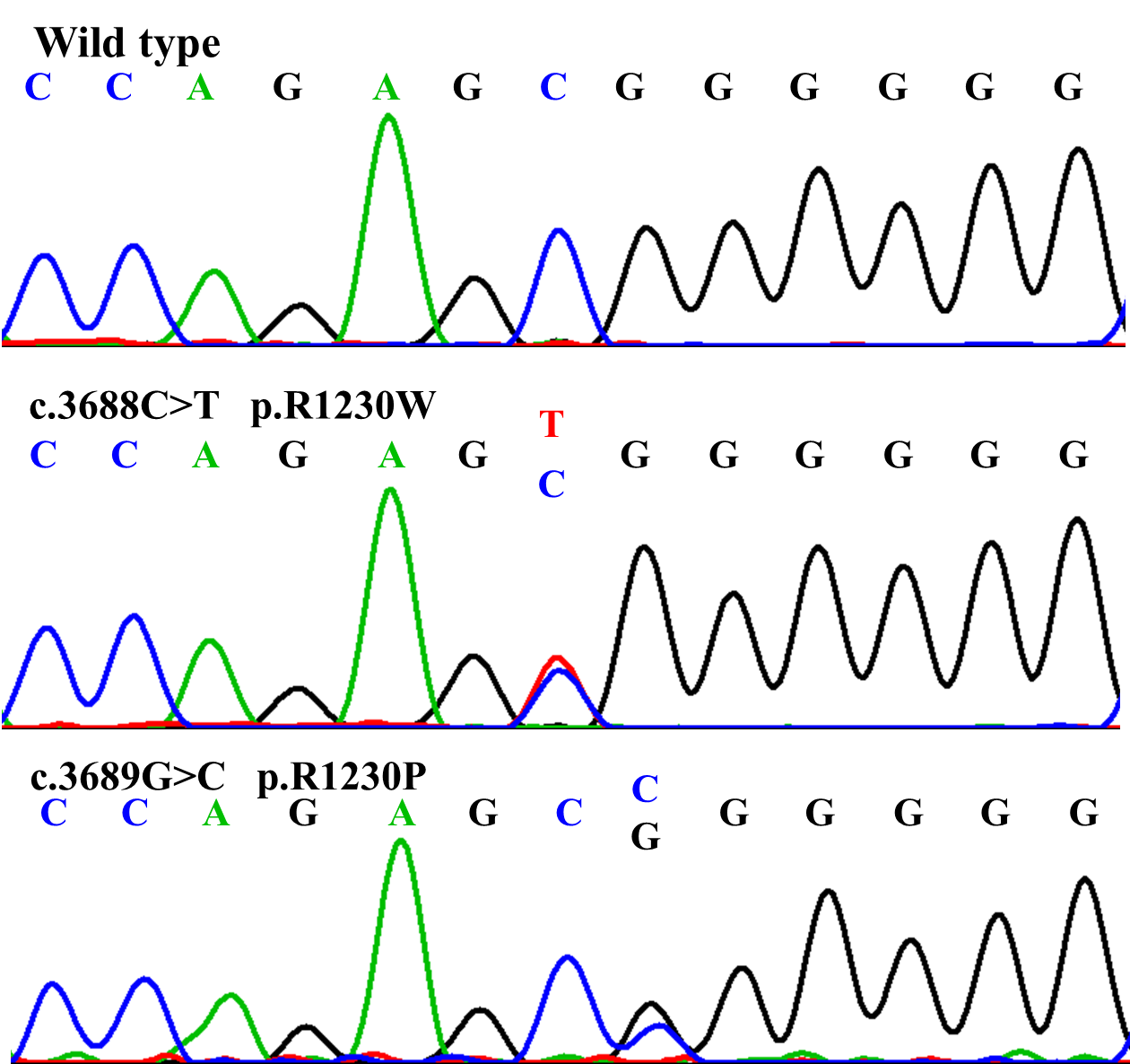
**

**F**

**
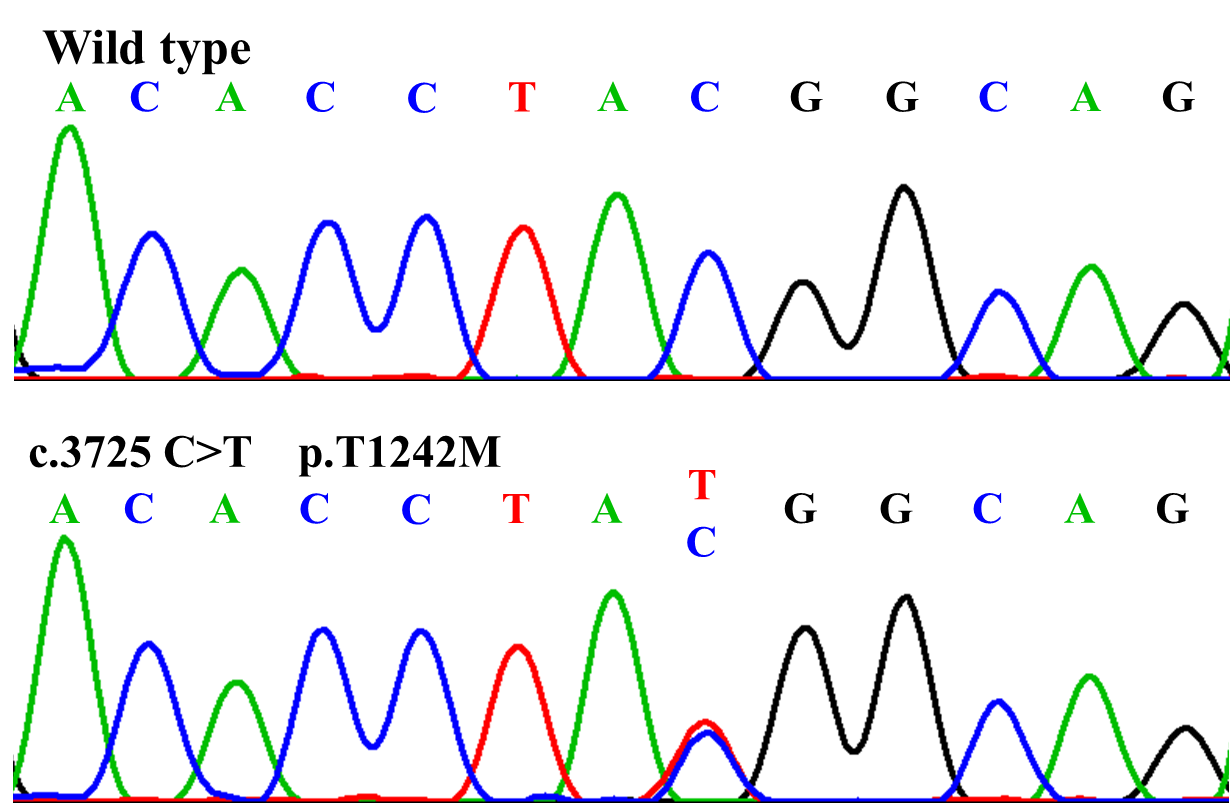
**

**G**

**
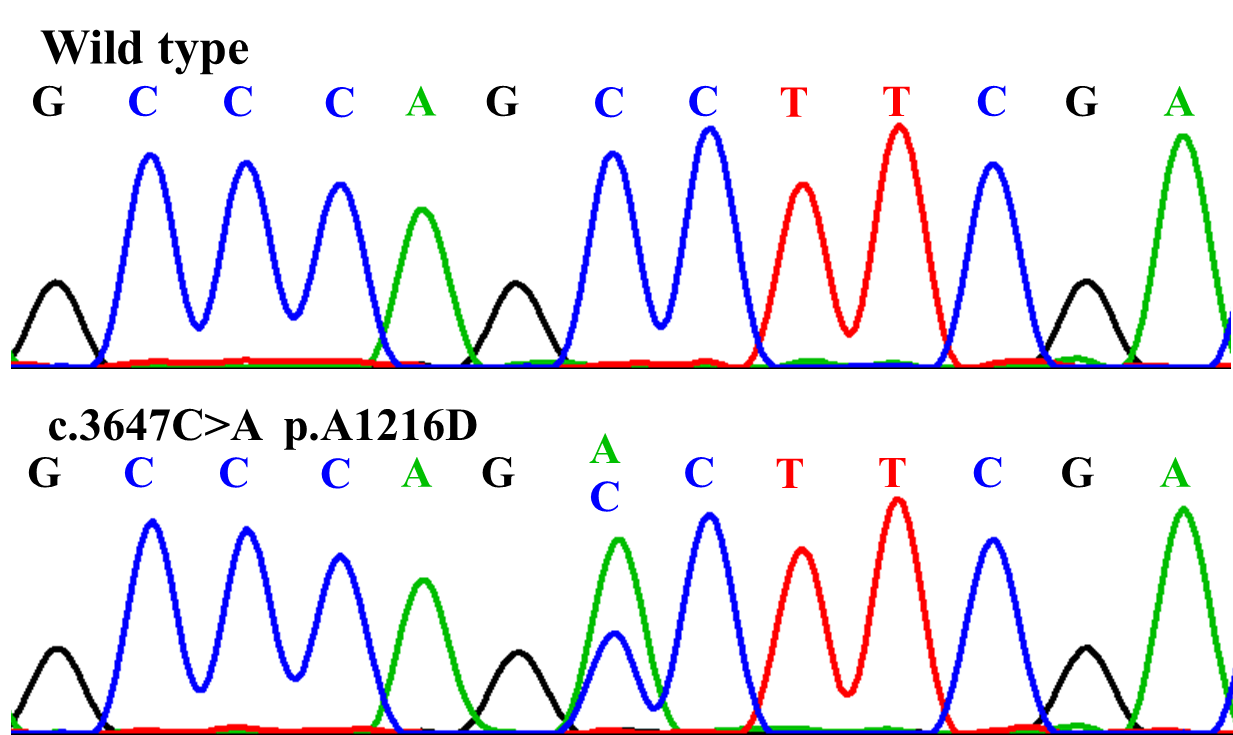
**

**H**

**
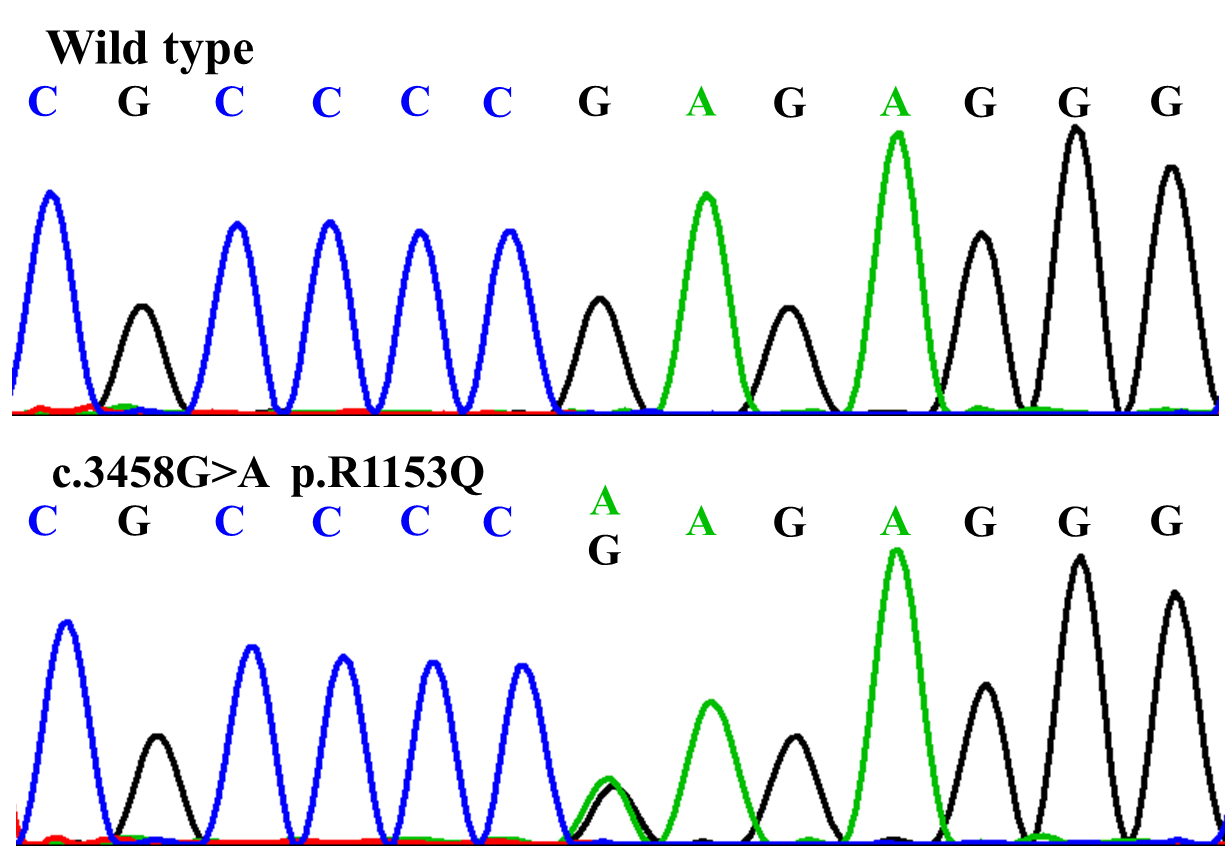
**
